# Physicians’ opinions on and practical experiences with palliative sedation therapy in children: an international survey in five European countries

**DOI:** 10.1186/s12904-025-01863-7

**Published:** 2025-10-16

**Authors:** Hanna Fischer, Deborah Gubler, Arjanne M. van Norel, Eva Bergsträsser, Joana Mendes, Ana Lacerda, Erna Michiels, Jitka Kosikova, Kristýna Poláková, Sara Depulpaep, Yoni Aelvoet, Laure Dombrecht, Kim Beernaert

**Affiliations:** 1 School of Human Medicine, University of Zurich Faculty of Medicine, Zurich, Switzerland; 2 Paediatric Palliative Care and Children’s Research Centre, University Children’s Hospital Zurich, Zurich, Switzerland; 3 Department of Anaesthesiology and Pain Medicine, Princess Máxima Center for Paediatric Oncology, Utrecht, The Netherlands; 4 Nursing School of Lisbon, Nursing Research Innovation and Development Centre of Lisbon, Lisbon, Portugal; 5 Department of Paediatrics, Portuguese Institute of Oncology, Lisbon Centre, Portugal; 6 Department of Neuro-oncology and Quality of Life, Princess Máxima Center for Paediatric Oncology, Utrecht, The Netherlands; 7 The Czech Society of Palliative Medicine, Czech Medical Association of J.E. Purkyne, Prague, Czech Republic; 8 Institute Pallium, Prague, Czech Republic; 9 Paediatric Department, Ghent University Hospital, Ghent, Belgium; 10 End-of-Life Care Research Group, Vrije Universiteit Brussel (VUB) & Ghent University, Ghent, Belgium

**Keywords:** Palliative sedation therapy, Palliative care, Paediatrics, End of life

## Abstract

**Background:**

Palliative sedation therapy (PST) is used in the end of life (EOL) phase for terminally ill patients to alleviate refractory symptoms by lowering consciousness. This applies to both adults and children. No international comparative studies on the practice of paediatric PST exist, although such knowledge is needed for the development of guidelines and a broader discussion, including ethical aspects.

**Methods:**

An international retrospective cross-sectional survey in five European countries (Belgium, Czechia, Netherlands, Portugal, Switzerland) was performed. Questionnaires were distributed via email. Topics of the questionnaire concerned paediatric PST practices and opinions/attitudes about ethical discussion points. Data was analysed using descriptive analyses, chi-square-tests, and ANOVA tests.

**Results:**

Three hundred and forty-eight physicians completed the questionnaire (127 Belgium, 78 Czechia, 45 Netherlands, 57 Portugal, 21 Switzerland). 32% of respondents were specialized in general paediatrics. Most respondents use midazolam (85%) and opioids (78%) for PST. Parents are almost always involved in decision-making (93%), whereas competent children are involved to a lesser extent (77%). There is a greater agreement that refractory physical symptoms are an indication for PST (99%) than refractory psychological symptoms (69%). Most participants (63%) agreed to withdraw artificial hydration/nutrition in the last hours/days of life. Only 37% disagreed that PST shortens the dying process. We found significant differences in answers by country, speciality, workplace, experience and palliative care training.

**Conclusions:**

Nearly all of the 348 physicians across Belgium, Czechia, Netherlands, Portugal and Switzerland agreed that refractory physical symptoms are an indication for PST and, in practice, use midazolam and opioids most frequently. Variations exist in medication choices, decision-making processes, intentions for sedation, and attitudes towards ethical considerations. These findings suggest that educational, cultural, legal, and healthcare system differences shape the approach to palliative sedation.

**Supplementary Information:**

The online version contains supplementary material available at 10.1186/s12904-025-01863-7.

## Background

Palliative sedation therapy (PST) is a possible means to alleviate otherwise unbearable and refractory symptoms at the end of life (EOL). It can be carried out intermittently or continuously until death [1]. The practice of PST is the induced lowering of consciousness of a terminally-ill child with a life-limiting condition until death [2]. To date, no study has been conducted to describe medical practices and physicians’ opinions in a paediatric context at an international level.

Children who receive PST most frequently suffer from one or more of the following symptoms: pain, dyspnoea, agitation, anxiety, and seizures [3–8]. Psycho-existential suffering is difficult to assess in children, and the literature concerning the topic of psychological distress leading to PST is scarce [6]. As such, there is no consensus on whether it qualifies for PST in the absence of refractory physical symptoms [9]. Decision-making in paediatrics includes the assessment of the capacity and competence of children and surrogate decision makers [10]. Even children with adequate cognitive capacity are not always fully included in decisions about PST [11, 12]. The decision whether to initiate PST is followed by the aim of PST and the choice of the most appropriate medication. Benzodiazepines and opioids are the most common medications used for PST [6, 8, 13–15] while barbiturates are used occasionally [3]. Propofol, dexmedetomidine, ketamine or neuroleptics are prescribed even less frequently [3, 8]. Physicians are found to have different opinions on the withdrawal of artificial nutrition and fluids (WANF) during PST [9]. Additionally, there are ethical issues, for instance contravening the autonomy of the child [16].

There are no international guidelines for PST. To our knowledge, only the Netherlands have published national guidelines on PST which are, in their full form, available only in Dutch [17]. There is a need for broader knowledge on and concepts for PST and its indications, including the role of psychological distress, ethics and the decision-making process, the appropriate medication, and the withdrawal of artificial nutrition/hydration.

In this international study, we describe physicians’ practices and opinions on PST for terminally ill children in Belgium, Czechia, the Netherlands, Portugal, and Switzerland. As such, this study seeks to contribute to the discussion about PST and to inform European and national guideline development for PST.

## Methods

### Study design

An international retrospective cross-sectional survey study distributed in Belgium, Czechia, the Netherlands, Portugal and Switzerland, based on the survey of Heijltjes et al. [18], which was performed in a similar context than ours, but addressing adult PST.

### Setting and participants

We approached physicians working in general and specialised paediatrics experienced in caring for terminally-ill children. Target participants were those who had been involved in EOL care of at least one paediatric patient in the past three years. The research teams of the participating countries distributed the survey separately via email to professionals by using personal contacts and/or mailing lists of scientific associations. A nonresponse survey assessed refusals due to inexperience with PST. To minimise nonresponses, a follow-up reminder was sent 2–4 weeks after the initial invitation. We sent out Emails to the participating countries between October 2023 and February 2025, one country at a time, with each country having a response window of approximately 1.5 months. To avoid duplicate responses, we asked the respondent’s initials followed by the last 2 digits of their birth year followed by the last 2 letters of their last name. We excluded 6 filled out questionnaires due to doubles.

### Questionnaires

Our questionnaire was developed based on existing international surveys on PST in children [12], neonates [19] and adults [18]. It was reviewed by members of the European Association for Palliative Care (EAPC) Children and Young People Reference Group, and distributed through Lime Survey [20]. The research teams of the different countries translated it, with the option to discuss possible semantic doubts with the questionnaire’s creators. The questionnaire consists of four parts: participant’s medical practices regarding PST, their opinions and attitudes about these practices, sociodemographic information, and their proposed medical decision-making process for a chosen vignette case. Respondents could only fill out the part on medical practice if they had answered “yes” to the question whether they had ever provided PST.

In the question about whether respondents start medication at a sufficiently high level or start low and increase the dosage gradually, the exact dosages were left to individual interpretation.

### Statistical analysis

For questions about frequency, we summarized the answers “always” and “often” as “yes = 1” and the options “never” and “rarely” as “no=−1”. For questions about agreement with statements, we summarized the options “agree” and “rather agree” as “agree = 1” and the options “disagree” and “rather disagree” as “disagree=−1”. We coded the options “sometimes” and “neutral/not sure” as “0”. This regrouping should improve readability. If respondents did not answer to all questions, any missing answers were excluded from further analysis.

We excluded the answers of neonatologists to the question about the involvement of competent and partly competent children in decision-making, as neonates never fulfil these criteria.

We performed the descriptive analysis strategies of calculating numbers, percentages and confidence intervals via chi-square tests for calculating the significance. Statistical significance was set at a *p*-value < 0.05. We searched for differences between sociodemographic factors and answers regarding practices and opinions. IBM SPSS statistics Version 29.0.2.0 was used [16].

## Results

### Demographics

We received 348 completed questionnaires (127 Belgium, 78 Czechia, 45 Netherlands, 57 Portugal, 21 Switzerland). 32% of respondents had a specialisation in general paediatrics, and their mean experience as a physician was 20 years (range 0–51 years, median 20 years, standard deviation 10.8). 40% of respondents had received palliative care education/training of ≥ 1 day. 52% did not receive a palliative care education/training. Only 23% had no experience with PST. Table 1 shows the detailed demographics of the participants in this study.

**Table 1:** Demographics

	Total (*n* = 348)	Belgium (*n* = 127)	The Netherlands (*n* = 45)	Switzerland (*n* = 41)	Czech (*n* = 78)	Portugal (*n* = 57)
Gender* Female Male Prefer not to say	234 (72%) 86 (27%) 5 (1.5%)	91 (75%) 29 (24%) 2 (2%)	31 (74%) 8 (19%) 3 (7%)	22 (63%) 13 (37%) 0 (0%)	44 (60%) 29 (40%) 0 (0%)	46 (87%) 7 (13%) 0 (0%)
*Age Group* < 30 years 30–39 years 40–49 years > 50 years	12 (4%) 86 (27%) 96 (30%) 131 (40%)	6 (5%) 44 (36%) 29 (24%) 43 (35%)	0 (0%) 7 (17%) 14 (33%) 21 (50%)	0 (0%) 2 (6%) 17 (49%) 16 (46%)	5 (7%) 17 (23%) 21 (29%) 30 (41%)	1 (2%) 16 (30%) 15 (28%) 21 (40%)
*Clinical Speciality* Paediatric Palliative Medicine/Care Paediatrics (general) Paediatric Intensive Care Paediatric Oncology Paediatric Neurology Paediatric Cardiology Paediatric Metabolic Diseases Paediatric Anesthesiology Neonatal Intensive Care Adult Palliative Care General/Family Practice Other	22 (7%) 104 (32%) 32 (10%) 36 (11%) 17 (5%) 2 (1%) 4 (1%) 1 (< 1%) 60 (19%) 5 (2%) 23 (7%) 19 (6%)	2 (2%) 41 (34%) 5 (4%) 17 (14%) 10 (8%) 1 (1%) 2 (2%) 0 (0%) 22 (18%) 0 (0%) 11 (9%) 11 (9%)	4 (10%) 8 (19%) 6 (14%) 4 (10%) 1 (2%) 0 (0%) 1 (2%) 0 (0%) 7 (17%) 0 (0%) 9 (21%) 2 (5%)	10 (29%) 4 (11%) 4 (11%) 3 (9%) 0 (0%) 0 (0% 0 (0%) 0 (0%) 13 (37%) 0 (0%) 0 (0%) 1 (3%)	3 (4%) 30 (41%) 12 (16%) 3 (4%) 5 (7%) 0 (0%) 0 (0%) 0 (0%) 8 (11%) 5 (7%) 3 (4%) 4 (6%)	3 (6%) 23 (43%) 5 (9%) 9 (17%) 1 (2%) 1 (2%) 1 (2%) 1 (2%) 8 (15%) 0 (0%) 0 (0%) 1 (2%)
Years of work experience as a doctor: mean (range, median)	19.93 (0–50, 20)	18.64 (2–40, 17)	20.88 (5–43, 23)	18.74 (0–34, 20)	20.58 (0–50, 20)	21.98 (4–51, 20)
*Religion* No religion Christianity Judaism Islam Other Prefer not to say	148 (46%) 150 (46%) 2 (1%) 1 (< 1%) 10 (3%) 14 (4%)	54 (44%) 58 (48%) 0 (0%) 0 (0%) 9 (7%) 1 (1%)	27 (64%) 13 (31%) 1 (2%) 0 (0%) 1 (2%) 0 (0%)	12 (34%) 20 (57%) 0 (0%) 0 (0%) 0 (0%) 3 (9%)	39 (53%) 27 (37%) 1 (1%) 0 (0% 0 (0%) 6 (8%)	16 (30%) 32 (60%) 0 (0%) 1 (2%) 0 (0%) 4 (8%)
*Specialist palliative care training* training of < 1 day training of 1–3 days training of > 3 days No training	27 (8%) 55 (17%) 74 (23%) 169 (52%)	10 (8%) 11 (9%) 15 (12%) 86 (71%)	4 (10%) 10 (24%) 13 (31%) 15 (36%)	1 (3%) 4 (11%) 19 (54%) 11 (31%)	8 (11%) 13 (18%) 13 (18%) 39 (53%)	4 (8%) 17 (32%) 14 (26%) 18 (34%)
*Number of paediatric patients cared for in the past year * None 1–3 patients 4-6 patients > 6 patients	89 (27%) 142 (44%) 45 (14%) 49 (15%)	42 (34%) 49 (40%) 11 (9%) 20 (16%)	5 (12%) 24 (57%) 7 (17%) 6 (14%)	1 (3%) 16 (46%) 8 (23%) 10 (29%)	32 (44%) 23 (31%) 12 (16%) 6 (8%)	9 (17%) 30 (57%) 7 (13%) 7 (13%)
Ever provided the continuous use of sedatives as a means to alleviate severe suffering in the last hours to days of life Yes No	268 (77%) 80 (23%)	97 (76%) 30 (24%)	42 (93%) 3 (7%)	35 (85%) 6 (15%)	45 (58%) 33 (42%)	49 (86%) 8 (14%)

*The option “other” was not chosen by any of the respondents

### Practice

Table 2 provides details of results concerning medications used for PST, decision-making, and intention for PST.

**Table 2:** Practice

	Total	Countries						Training				
Topic	Total (*N* = 268)	Belgium (*N* = 97)	Netherlands (*N* = 42)	Switzerland (*N* = 35)	Czechia (*N* = 45)	Portugal (*N* = 49)	p-value countries	No Palliative Training (*N* = 114)	Palliative Training of < 1 day (*N* = 23)	Palliative Training of 1–3 days (*N* = 51)	Palliative Training of > 3 days (*N* = 68)	p-value training
Medication used for PST												
Midazolam n (% of the column)	228 (85%)	88 (91%)	42 (100%)	26 (74%)	35 (78%)	37 (76%)	< 0.001	94 (84%)	16 (70%)	41 (80%)	64 (96%)	0.007
Opioids (with intent to provide sedation) n (% of the column)	209 (78%)	76 (78%)	27 (64%)	31 (89%)	36 (80%)	39 (80%)	0.140	90 (80%)	20 (87%)	46 (90.2%)	43 (64%)	0.004
Dexmedetomidine n (% of the column)	37 (14%)	9 (9%)	3 (7%)	7 (20%)	14 (31%)	4 (8%)	0.005	12 (11%)	3 (13%)	9 (18%)	11 (16%)	0.587
Propofol n (% of the column)	35 (13%)	15 (16%)	8 (19%)	4 (11%)	6 (13%)	2 (4%)	0.176	9 (8%)	4 (17%)	11 (22%)	11 (16%)	0.091
Levomepromazine/chlorpromazine n (% of the column)	23 (9%)	3 (3%)	14 (33%)	0 (0%)	2 (4%)	4 (10%)	< 0.001	2 (2%)	1 (4%)	9 (18%)	8 (12%)	0.002
Barbiturate n (% of the column)	18 (7%)	5 (5%)	4 (10%)	2 (6%)	6 (13%)	1 (2%)	0.363	8 (7%)	1 (4%)	4 (8%)	5 (8%)	0.949
Haloperidol n (% of the column)	14 (5%)	6 (6%)	1 (2%)	2 (6%)	4 (9%)	1 (2%)	0.501	5 (5%)	1 (4%)	2 (4%)	5 (8%)	0.810
Others* n (% of the column)	12 (5%)	2 (2%)	3 (7%)	3 (9%)	3 (7%)	1 (2%)	0.329	4 (4%)	1 (4%)	2 (4%)	4 (6%)	0.902
Participation in medical decision-making**												
A child who is competent N (% of column yes) N (% of column no)	160 (77%) 24 (12%)	59 (79%) 7 (9%)	27 (79%) 2 (6%)	18 (90%) 2 (10%)	30 (79%) 4 (11%)	26 (62%) 9 (21%)	0.153	56 (75%) 11 (15%)	13 (68%) 2 (11%)	33 (75%) 5 (11%)	48 (83%) 4 (7%)	0.71
A child who is not fully competent (due to age or disability) N (% of column yes) N (% of column no)	56 (27%) 86 (41%)	23 (31%) 21 (28%)	15 (44%) 10 (30%)	9 (45%) 4 (20%)	6 (16%) 22 (58%)	3 (7%) 29 (70%)	< 0.001	17 (23%) 32 (43%)	3 (16%) 10 (53%)	9 (21%) 22 (50%)	24 (41%) 18 (31%)	0.139
A child who is incompetent (due to age or disability) N (% of column yes) N (% of column no)	20 (8%) 212 (80%)	12 (12%) 73 (75%)	3 (7%) 29 (71%)	4 (12%) 24 (73%)	1 (2%) 41 (91%)	0 (0%) 45 (92%)	0.009	7 (6%) 94 (84%)	2 (9%) 18 (78%)	2 (4%) 42 (82%)	6 (9%) 51 (76%)	0.845
The parents N (% of column yes) N (% of column no)	247 (93%) 9 (3%)	93 (96%) 0 (0%)	40 (98%) 1 (2%)	31 (91%) 2 (6%)	42 (93%) 2 (4%)	41 (84%) 4 (8%)	0.046	106 (95%) 3 (3%)	19 (83%) 2 (9%)	48 (94%) 2 (4%)	64 (96%) 2 (3%)	0.626
The siblings N (% of column yes) N (% of column no)	26 (10%) 187 (70%)	9 (9%) 64 (66%)	6 (15%) 21 (51%)	5 (15%) 24 (71%)	4 (9%) 36 (80%)	2 (4%) 42 (86%)	0.019	7 (6%) 85 (76%)	2 (9%) 15 (65%)	4 (8%) 38 (75%)	10 (15%) 41 (61%)	0.412
Intention**												
relieve patient suffering N (% of column yes) N (% of column no)	261 (99%) 0 (0%)	94 (97%) 0 (0%)	41 (100%) 0 (0%)	33 (100%) 0 (0%)	45 (100%) (0%)	48 (98%) 0 (0%)	0.291	112 (100%) 0 (0%)	22 (96%) 0 (0%)	51 (100%) 0 (0%)	66 (99%) 0 (0%)	0.193
relieve parental suffering N (% of column yes) N (% of column no)	114 (43%) 73 (28%)	33 (34%) 28 (29%)	5 (12%) 23 (56%)	11 (33%) 7 (21%)	30 (67%) 7 (16%)	35 (71%) 8 (16%)	< 0.001	52 (46%) 27 (24%)	10 (44%) 4 (17%)	23 (45%) 17 (33%)	24 (36%) 20 (30%)	0.458
relieve healthcare provider suffering N (% of column yes) N (% of column no)	20 (8%) 198 (75%)	2 (2%) 77 (79%)	1 (2%) 37 (90%)	2 (6%) 25 (76%)	6 (13%) 27 (60%)	9 (18%) 32 (65%)	0.003	6 (5%) 83 (74%)	4 (17%) 13 (57%)	6 (12%) 38 (75%)	3 (5%) 56 (84%)	0.128
To decrease the patient’s consciousness N (% of column yes) N (% of column no)	56 (21%) 127 (48%)	22 (23%) 51 (53%)	9 (22%) 19 (46%)	3 (9%) 17 (52%)	11 (24%) 18 (40%)	11 (22%) 22 (45%)	0.551	19 (17%) 59 (53%)	10 (44%) 7 (30%)	10 (20%) 26 (51%)	13 (19%) 31 (46%)	0.235
To induce unconsciousness N (% of column yes) N (% of column no)	18 (7%) 197 (74%)	10 (10%) 68 (70%)	1 (2%) 33 (81%)	1 (3%) 28 (85%)	4 (9%) 31 (69%)	2 (4%) 37 (76%)	0.513	7 (6%) 88 (79%)	6 (26%) 13 (57%)	2 (4%) 44 (86%)	3 (5%) 46 (69%)	0.012
To shorten the dying process N (% of column yes) N (% of column no)	23 (9%) 185 (70%)	15 (16%) 48 (50%)	0 (0%) 34 (83%)	1 (3%) 28 (85%)	5 (11%) 32 (71%)	2 (4%) 43 (88%)	< 0.001	17 (15%) 68 (61%)	1 (4%) 12 (52%)	0 (0%) 43 (84%)	4 (6%) 55 (82%)	< 0.001

Missings are in all columns ≤ 5%

Respondents could only fill out this part on medical practices if they answered yes to question if they have ever provided PST (77% of the respondents)

*For example: Ketamin, Chloralhydrat etc

**We only report the % of “yes” and “no” and not the % of neutrals due to better readability

### Medication

Overall, midazolam (85%) and opioids (78%) are most frequently used for PST. Dexmedetomidine (14%) and propofol (13%); levomepromazine/chlorpromazine (9%), barbiturates (7%) and haloperidol (5%) are used occasionally. Compared with less experienced physicians, those who had cared for > 6 children at the EOL in the last year used midazolam (98%, p-value = 0.003) and dexmedetomidine (26%, *p*-value = < 0.001) significantly more often. Paediatric intensive care physicians used propofol (44%, *p* = < 0.001), dexmedetomidine (34%, p-value = < 0.001) and levomepromazine (22%, *p*-value = 0.004) significantly more frequently than the other specialities. Paediatric palliative medicine specialists also used dexmedetomidine (25%, *p*-value = < 0.001) and levomepromazine/chlorpromazine (20%, *p*-value = 0.004) significantly more often.

Most respondents (65%) started with a lower dose and slowly increased medication, whereas 29% started at a presumably sufficiently high level. Physicians who had cared for ≥ 4 children at the EOL in the last year were significantly more likely to start at a higher dose (40%, *p*-value = 0.013) than those less experienced (24%).

### Decision-making

In 93% of the cases, physicians involved parents in decision-making about PST. Competent children were involved in 77%. Not fully competent children were involved by 27% of the physicians, and incompetent children by 8%. Siblings were rarely involved (10%). The involvement of not fully competent children was significantly lower in Czechia and Portugal (16% and 7%, *p*-value = < 0.001) but had a tendency to be higher when physicians had a training in palliative care > 3 days (41%, *p*-value = 0.139).

### Intention

Respondents most often reported reducing patient suffering as an intention for PST (99%). To a lesser extent, reducing parental suffering (43%), reducing consciousness (21%), shortening the dying process (9%), reducing healthcare professionals’ (HCP) suffering (8%) and inducing unconsciousness (7%) were reported.

In Czechia and Portugal, physicians cited reducing parental (67% and 71%, *p*-value = < 0.001) and HCP (13% and 18%, *p*-value = 0.003) suffering significantly more often as their intentions than in other countries (12–34% and 2–8%, respectively). Physicians working in a nonhospital setting insignificantly more frequently agreed that the goal of PST is only attained when the patient is unconscious (27%, *p*-value = 0.051) than did physicians working in a hospital (11%).

### Opinions and attitudes

In this section we report our results of physicians’ attitudes towards the indications of PST, the withdrawal of artificial hydration/nutrition during PST, and the question if PST shortens the dying process. See Table 3 for more detailed results.

**Table 3:** Opinions

	Total	Countries						Training				
Topic	Total (*N* = 348)	Belgium (*N* = 127)	Netherlands (*N* = 45)	Switzerland (*N* = 41)	Czechia (*N* = 78)	Portugal (*N* = 57)	p-value countries	No Palliative Training (*N* = 169)	Palliative Training of < 1 day (*N* = 27)	Palliative Training of 1–3 days (*N* = 55)	Palliative Training of > 3 days (*N* = 74)	p-value training
Indication												
The continuous use of sedatives to alleviate severe physical suffering in the last hours to days of life is an acceptable medical practice. N (% of column yes) N (% of column no)	328 (99%) 2 (1%)	121 (99%) 0 (0%)	43 (100%) 0 (0%)	36 (97%) 1 (3%)	74 (99%) 1 (1%)	54 (100%) 0 (0%)	0.581	168 (99%) 1 (1%)	26 (96%) 0 (0%)	55 (100%) 0 (0%)	73 (99%) 1 (1%)	0.370
The continuous use of sedatives to alleviate severe psycho-existential suffering (in the absence of physical symptoms) in the last hours to days of life is an acceptable medical practice. N (% of column yes) N (% of column no)	227 (69%) 21 (6%)	67 (55%) 7 (6%)	26 (61%) 3 (7%)	25 (69%) 3 (8%)	63 (84%) 3 (4%)	46 (85%) 5 (10%)	< 0.001	113 (67%) 13 (8%)	21 (78%) 0 (0%)	41 (75%) 2 (4%)	48 (65%) 6 (8%)	0.398
The continuous use of sedatives to alleviate severe physical suffering for patients who are expected to live for at least several weeks is an acceptable medical practice. N (% of column yes) N (% of column no)	257 (78%) 21 (6%)	103 (84%) 3 (3%)	24 (56%) 8 (19%)	26 (72%) 2 (6%)	63 (84%) 4 (5%)	41 (76%) 4 (7%)	0.011	141 (83%) 7 (4%)	22 (82%) 0 (0%)	45 (82%) 2 (4%)	45 (61%) 11 (15%)	0.003
The continuous use of sedatives to alleviate severe psycho-existential suffering (in the absence of physical symptoms) for patients who are expected to live for at least several weeks is an acceptable medical practice. N (% of column yes) N (% of column no)	143 (44%) 58 (18%)	46 (38%) 25 (21%)	10 (23%) 14 (33%)	15 (42%) 4 (11%)	42 (56%) 7 (9%)	30 (57%) 8 (15%)	0.003	80 (47%) 29 (17%)	11 (41%) 1 (4%)	28 (51%) 3 (6%)	22 (30%) 23 (31%)	< 0.001
Shortening the dying process												
PST in the last hours to days of life shortens the dying process. N (% of column yes) N (% of column no)	95 (29%) 120 (37%)	52 (43%) 24 (20%)	10 (23%) 17 (40%)	9 (26%) 11 (31%)	17 (23%) 32 (43%)	7 (13%) 36 (68%)	< 0.001	60 (36%) 45 (27%)	6 (22%) 10 (37%)	15 (27%) 23 (42%)	14 (19%) 40 (54%)	0.004
PST in the last hours to days can be difficult to distinguish from euthanasia. N (% of column yes) N (% of column no)	59 (18%) 221 (68%)	32 (26%) 70 (57%)	5 (12%) 33 (77%)	5 (14%) 23 (66%)	13 (18%) 49 (66%)	4 (8%) 46 (87%)	0.011	40 (24%) 97 (57%)	6 (22%) 15 (56%)	3 (6%) 46 (84%)	10 (14%) 61 (82%)	< 0.001
Withdrawal of artificial hydration/nutrition												
The routine withdrawal of artificial hydration/nutrition while providing PST in the last hours to days of life is acceptable. N (% of column yes) N (% of column no)	207 (63%) 76 (23%)	86 (71%) 23 (19%)	41 (95%) 1 (2%)	22 (63%) 6 (17%)	32 (43%) 26 (35%)	26 (49%) 20 (38%)	< 0.001	100 (59%) 42 (25%)	12 (44%) 11 (41%)	36 (66%) 14 (26%)	57 (77%) 9 (12%)	0.03
The routine withdrawal of artificial hydration/nutrition while providing PST in patients who are expected to live for at least several weeks is acceptable. N (% of column yes) N (% of column no)	79 (24%) 189 (58%)	35 (29%) 63 (52%)	23 (54%) 10 (23%)	8 (23%) 20 (57%)	9 (12%) 54 (73%)	4 (8%) 42 (79%)	< 0.001	35 (21%) 103 (61%)	3 (11%) 20 (74%)	16 (29%) 27 (49%)	24 (32%) 38 (51%)	0.167

Missings are ≤ 6% in all columns except for Switzerland with 10–15% missings

We only report the % of “yes” and “no” and not the % of neutrals due to better readability

### General

95% of respondents agreed that, after being fully informed, a competent child with severe suffering has the right to demand the continuous use of sedatives in the last hours or days of life. There was a high agreement (92%) with the same statement regarding the parents.

### Indications for PST

Regardless of the physical or psychological nature of symptoms, an expected survival of hours to days led to more agreement on the indication for PST (69% [psychological symptoms] and 99% [physical symptoms]) than an expected survival of weeks (44% [psychological symptoms] and 78% [physical symptoms]).

Withdrawal of artificial hydration and/or nutrition.

There was an agreement of 63% to withdraw artificial hydration/nutrition in the last hours to days, whereas only 24% agreed to do so in the last weeks. Across all countries, those physicians with palliative care training of > 3 days significantly more often agreed with the withdrawal in the last hours/days (77%, p-value = 0.03). Physicians in the Netherlands had a significantly greater acceptance (95%, p-value = < 0.001) of this withdrawal in the last hours, and this was the only country with a tendency to agree (54%, p-value = < 0.001) to withdrawal in the last few weeks. On the other hand, physicians in Czechia showed by far the lowest agreement to withdrawal in the last hours to days (43%, p-value = 0.001) and in Portugal in the last weeks (8%, p-value = < 0.001).

### Shortening of the dying process

29% of the respondents agreed that PST shortens the dying process, and 18% stated that PST is hard to distinguish from euthanasia. With a palliative care training > 3 days, physicians disagreed significantly more strongly that it shortens the dying process (54%, p-value = 0.001) and found it less difficult to distinguish from euthanasia (14%, p-value = 0.001). Compared to other specialities, neonatologists found it significantly more difficult to distinguish between PST and euthanasia (35%, p-value = 0.011). Compared to other countries (13–23%), Belgian respondents agreed significantly more often that PST shortens the dying process (43%, p-value = 0.001).

## Discussion

This international survey study shows how PST is used in children and that certain overarching trends are observed, such as the predominant use of midazolam and opioids. Significant differences exist between practices and opinions of physicians depending on their sociodemographic background. This survey aims to shed light on this situation and to offer suggestions for education and the development of national and international paediatric PST guidelines.

### Medication

The first-choice medication for PST in our survey was midazolam, which is in line with the literature [6, 8, 11, 14, 17]. Compared with studies on adult PST [2, 18], we found a more frequent use of opioids as medication for sedation. Whereas international guidelines for adult PST state not to use opioids for PST [21], the Dutch guidelines on paediatric PST mention opioids as a possible option for sedation in case of pain and dyspnoea [17]. Opioids might have a bigger importance in PST of neonates because benzodiazepines are not generally recommended to be used in this age group [22].

We observed that more experienced physicians more frequently used dexmedetomidine, which has analgesic properties in addition to its sedating effect [23]. Levomepromazine, used more often by the Netherlands than by the other countries, has a co-analgetic and anti-emetic effect [24]. Propofol was seldomly used, and mainly by paediatric intensive care specialists. As Miele et al. and Anghelescu et al. reported [25, 26], the benefit of propofol in PST is the potential opioid-sparing effect of propofol. We observed differences in medication habits depending on the physician’s speciality and in different countries. These differences in additional medication choices probably reflect among others national protocols, cultural habits, physician’s specialisation, and physician’s training as well as institution’s rules for the use of specific medications such as propofol or dexmedetomidine. A discussion at an international level would be essential to develop a general PST guideline for children. Then, in a second step, this guideline would have to be adapted to the applicable rules at the respective national level.

### Ethical considerations

Medical ethics are based on the four principles of Beauchamp and Childress [27]: “ (1) respect for autonomy (a norm of respecting the decision-making capacities of autonomous persons) (2), nonmaleficence (a norm of avoiding the causation of harm) (3), beneficence (a group of norms for providing benefits and balancing benefits against risks and costs), and (4) justice (a group of norms for distributing benefits, risks, and costs fairly)” [27]. We apply these principles in the following ethical discussion.

Indications and Intentions.

PST is used with the purpose to alleviate and treat unbearable and refractory symptoms that should be considered in a holistic perspective, involving physical but also psychological dimensions.

Most of the respondents agreed that refractory physical symptoms in the last hours/days are an indication for PST. Thus, PST can clearly be attributed to the principle of ‘beneficence’.

The indication is less clear when looking at refractory psychological symptoms at the EOL (e.g. anxiety, depression, but also behavioural alteration such as irritability, restlessness, aggressiveness) without refractory physical symptoms. Assessing the psychological symptom burden in young children or neonates may be difficult or almost impossible. The course of a specific psychological symptom may also be more difficult to predict and evaluating the point where all other therapies have been exhausted could be demanding. The interface between psychological and spiritual dimensions poses another challenge to interpretation and therapy. All this could explain the hesitation of respondents to agree that this could be an indication for PST. However, in some cases behavioural alterations, such as irritability and restlessness, e.g. in the context of delirium, may be even more burdensome than physical symptoms. Interprofessional teams with psychiatrists/psychologists could clarify and improve the decision-making process in this important symptom area.

The main intention of respondents doing PST was to reduce the patient’s suffering, nevertheless also the intention of reducing parental suffering was agreed to by 43% of the participants. We want to emphasize the importance of focussing on the wellbeing of the patient. Simultaneously, by giving the best care to the child, also the suffering of the parents can be reduced. Situations where the suffering of the parents is reduced by PST without thereby reducing the suffering of the child are ethically unsound as they do not fulfil the principal of beneficence [27].

### Decision-making

Parental involvement in decision-making was nearly universal, but the extent to which children were included varied. Consistent with the literature [11, 12], we found that children, even if competent, were not always included in decision-making. Competent children were involved in 77% of cases and not fully competent children in 27% of the cases though this was significantly lower in Czechia and Portugal (16% and 7%, respectively). These differences may reflect variations in how paediatric autonomy is perceived across cultures. Physicians with > 3 days training in palliative care were more likely to involve not fully competent children in decision-making (41% compared to 23% with no training). This suggests that education and experience play a key role in fostering inclusive decision-making processes. Even if the child is not fully competent in deciding for PST, its opinion, preferences and values, according to age, personality and developmental stage, should be considered in decision-making. Professional understanding of the difference between “capacity” and “competence” is mandatory to promote a child’s gradual autonomy [10].

### Withdrawal of artificial hydration/nutrition

Our findings indicate that physicians are more likely to agree with WANF in the final hours of life (63%) than in the last weeks (24%). This aligns with the intent to avoid artificially prolonging suffering while reducing respiratory and gastrointestinal distress. Studies indicate that most children experience increased comfort after WANF, with minimal distressing symptoms beyond dry lips or mouth [28].

However, WANF presents ethical dilemmas. Assessing hunger, thirst, and potential positive experiences in a state of reduced consciousness remains complex. Decision-making principles, such as those by Diekema et al. [29], may support physicians in navigating these challenges.

Country-specific differences highlight cultural and legal influences. Dutch physicians showed the highest acceptance, viewing WANF as part of the natural dying process, whereas Czech physicians had the lowest agreement, possibly due to legal concerns or limited experience in care limitation.

### Shortening the dying process

The literature describes the phenomenon of the “double effect” of PST, meaning that while PST does not have the primary intention to shorten the dying process, it is a possibility that is accepted to alleviate otherwise refractory suffering [11]. With a palliative training > 3 days palliative training, most respondents (82%) stated to see the difference between PST and euthanasia. This percentage was significantly higher compared to the group of respondents without palliative care training.

Our results suggest the importance of education and training in palliative care to improve ethical decision-making and understanding of PST as part of the internationally accepted concept of paediatric palliative care.

### Strengths and limitations

A key strength of our study is its international scope, allowing for cross-country comparisons and contributing to broader discussions on PST in children. It provides a foundation for knowledge exchange and guideline development.

However, the study has limitations. The survey distribution method prevented us to determine an exact response rate and introduced a possible selection bias due to also contacting the personal contacts of the researchers (leading to more respondents being experienced physicians, with palliative care training). This likely resulted in an overrepresentation of experienced physicians with a particular interest in PST and perhaps also palliative care training. However, their insights offer valuable depth, serving as a strong foundation for future research, policy discussions, and guideline development.

Furthermore, the question about prior use of PST did not explicitly specify paediatric cases. We retrospectively excluded respondents who stated in free-text comments that their experience was limited to adult PST. Given that the survey was targeted at paediatricians, we expect minimal bias from this limitation.

We did not include a question about the exact number of PST performed per physician, which would have helped to assess the experience of the participants more accurately. The differentiation between different amounts of palliative training was minimalistic.

Lastly, we did not collect data on exact medication dosages to minimize response burden, which would be of interest for future guideline development. In the question about dosage habits, we asked for general tendency and left exact dosages to the interpretation of the respondent, which might have introduced inter-respondent variability and bias in the responses. Nevertheless, as the analysis focused on identifying general trends—rather than absolute dose levels—across respondent groups, we believe this limitation did not substantially affect the main findings of the study. Addressing this in future studies could strengthen clinical recommendations.

## Conclusions

This international study provides valuable insights into how palliative sedation therapy is used in children, highlighting both common practices and significant variations across countries, seemingly influenced by the country of residence, specialty, workplace, training and experience.

PST is used to alleviate otherwise unbearable suffering in the EOL period. Our findings emphasize the importance of education for paediatricians working with children with serious illness. Our findings suggest that a longer palliative care training leads to more acceptance for the withdrawal of artificial nutrition/fluids, more involvement of not fully competent children in decision-making and a clearer differentiation between palliative sedation therapy and euthanasia. The found differences between countries and medical specialisations encourage cross-country and cross-specialities dialogue.

Further research is needed to help answer some conflicting questions including medication-choice, efforts to ensure children’s autonomy and participation in decision-making. This would contribute to the international dialogue and to subsequently establishing consensus-based guidelines on paediatric PST.

## Supplementary Information

Supplementary Material 1.

Supplementary Material 2.

Supplementary Material 3.

## Data Availability

The Lime Survey questionnaire can be found in in the appendix. The invitation letter for participation in the study and the anonymized data is available upon request to the corresponding author.

## Abbreviations

EOL: End of Life
PST: Palliative Sedation Therapy
HCP: Healthcare Professionals
WANF: Withdrawal of artificial nutrition/fluids
