# Supplementary material for: Physicians’ opinions on and practical experiences with palliative sedation therapy in children: an international survey in five European countries
Source: BMC Palliat Care. 2025 Oct 16;24:260. doi: 10.1186/s12904-025-01863-7 (PMC12532919; doi:10.1186/s12904-025-01863-7)
Supplement: Supplementary file 1 — Supplementary Material 1. [file 12904_2025_1863_MOESM1_ESM.docx]

Appendix Table 1 **Distribution**

| Distribution Strategy by Country | |
| --- | --- |
| Country | Distribution Strategy |
| Belgium | We recruited participants by mailing through newsletters and emails from associations of paediatricians (n=1300) and paediatric liaison teams (n=54) in Belgium. The questionnaire was further distributed through direct contact with major paediatric and neonatal departments, targeted mailing to experts in the field (n=22), and snowball sampling. Significant overlap between the different mailing lists and between mailing lists and targeted experts is possible but unable to determine. |
| Netherlands | We recruited participants by targeted mailing to experts in the field (n=49), and through the national knowledge centre for paediatric palliative care (n=21) where we ensured no overlap between both groups. Additionally, the invite to participate in the study was mentioned in a newsletter from the Dutch Association for Pediatrics (n=2427). |
| Switzerland | We recruited participants by distributing the survey through 3 organizations (Interessengemeinschaft Pädiatrische und Neonatologische Intensivmedizin (IG-PNI (n=107)), Equipe pédiatrique d’accompagnement en soins de support et de confort (PASSO, n=approx.13) and Swiss Society of Neonatology (SSN, n=172)). Further we sent it to 24 individual physicians through personal contact. The survey was further distributed via snowball sampling, but we were unable to get information from all the contacted professionals about their forwarding. Significant overlap between the different mailing lists and between mailing lists and targeted experts is possible but unable to determine. |
| Czech | We distributed the questionnaire with help of the Czech Medical Association of J.E. Purkyne and their societies. The number of unique email addresses, the questionnaire was sent to, cannot be identified due to the fact, that unknown number of physicians are members of more than one society. The estimated number of email addresses is 500. |
| Portugal | We disseminated the survey to the mailing lists of the Portuguese Association of Palliative Care (406 members) and the Portuguese Paediatric Society (1700 members). |
