# Supplementary material for: Physicians’ opinions on and practical experiences with palliative sedation therapy in children: an international survey in five European countries
Source: BMC Palliat Care. 2025 Oct 16;24:260. doi: 10.1186/s12904-025-01863-7 (PMC12532919; doi:10.1186/s12904-025-01863-7)
Supplement: Supplementary file 2 — Supplementary Material 2. [file 12904_2025_1863_MOESM2_ESM.pdf]

EAPC CHILDREN AND YOUNG PEOPLE REFERENCE GROUP
EUROPEAN SURVEY – PALLIATIVE SEDATION – PROPOSAL

The purpose of this questionnaire is to gain insight into physicians' practices and opinions in various European countries regarding paediatric palliative sedation, using the following definitions:

Paediatric palliative sedation is the use of sedative medication to relieve intolerable and refractory symptoms by reducing patient's consciousness in minors (<18 years) at the end of life.
Refractory symptoms are symptoms that 1) lead to severe suffering for the patient and 2a) cannot (timely) be treated with usual treatments or 2b) cannot be treated without unacceptable side effects.
Palliative sedation can be classified as continuous, acute and intermittent.
Continuous palliative sedation (CPS) is the use of sedation during the terminal phase, continued until the patient's death.
Acute palliative sedation (APS) may be needed to relieve suffering when a life-threatening situation suddenly occurs in which a patient is expected to die within minutes to at most a few hours.
Compared to CPS and APS, **intermittent palliative sedation (IPS)** involves administering sedation for a limited time with the intention to discontinue the medication and awakening the patient.

From here on out, when the term 'palliative sedation' is used, we will refer to the practice of paediatric continuous palliative sedation (CPS).

It is of course difficult to do justice to all the finer nuances of clinical practice in a short questionnaire. Please indicate those answers that most closely reflect your practice or opinion. Your answers will be valuable even if you have little clinical practice with continuous use of sedatives. All information will be aggregated, analysed and reported anonymously, to ensure anonymity of both respondents and their healthcare organisations. By answering this survey it is implied that you are giving your informed consent to participate voluntarily in our research.

Please tick the boxes at the answers of your choice.

Medical practices

The following questions concern palliative sedation as a means to alleviate severe suffering in the last hours to days of life in minors (<18 years old). For a definition, see page 1.

1. **Have you ever provided the continuous use of sedatives as a means to alleviate severe suffering in the last hours to days of life?**
   - ☐ Yes
   - ☐ No → go to question 19

2. **How many patients have you provided the continuous use of sedatives as a means to alleviate severe suffering in the last hours to days of life in the past 12 months?**
   - ☐ None
   - ☐ 1-3 patients
   - ☐ 4-6 patients
   - ☐ >6 patients

3. **What kind of medications do you commonly use to provide the continuous use of sedatives as a means to alleviate severe suffering in the last hours to days of life?** (Please choose all that apply)
   - ☐ Midazolam
   - ☐ Barbiturates
   - ☐ Propofol
   - ☐ Levomepromazine/chlorpromazine
   - ☐ Haloperidol
   - ☐ Opioids (with intent to provide sedation)
   - ☐ Dexmedetomidine
   - ☐ Other, namely: ________________
   - ☐ I don't know
   - ☐ I prefer not to say

How do you dose your medications when you provide the continuous use of sedatives as a means to alleviate severe suffering in the last hours to days of life in minors (<18 years old)?

	Never	Rarely	Some-times	Often	Always
4. I start low and gradually increase the dosage of the medications until the desired effect is reached.	☐	☐	☐	☐	☐
5. I start sufficiently high in order to reach the desired effect rapidly.	☐	☐	☐	☐	☐

Are children and/or their families involved in the decision-making when you provide the continuous use of sedatives as a means to alleviate severe suffering in the last hours to days of life of minors (< 18 years old)?

	Never	Rarely	Some-times	Often	Always
6. I involve a child who is **competent** to participate in medical decision-making.	☐	☐	☐	☐	☐
7. I involve a child who is **not fully competent** (due to age or disability) to participate in medical decision-making.	☐	☐	☐	☐	☐
8. I involve a child who is **incompetent** (due to age or disability) to participate in medical decision-making.	☐	☐	☐	☐	☐
9. I involve the parents in the medical decision-making.	☐	☐	☐	☐	☐
10. I involve brothers or sisters in the medical decision-making.	☐	☐	☐	☐	☐

When do you consider the goal of the palliative sedation to have been achieved?					
	Never	Rarely	Some-times	Often	Always
11. When the patient is comfortable (but not necessarily unconscious).	☐	☐	☐	☐	☐
12. When the patient is unconscious.	☐	☐	☐	☐	☐

What is your intention when you provide palliative sedation in the last hours to days of life?					
	Never	Rarely	Some-times	Often	Always
13. To relieve patient suffering	☐	☐	☐	☐	☐
14. To relieve parental suffering	☐	☐	☐	☐	☐
15. To relieve healthcare provider suffering	☐	☐	☐	☐	☐
16. To decrease the patient's consciousness	☐	☐	☐	☐	☐
17. To induce unconsciousness	☐	☐	☐	☐	☐
18. To shorten the dying process	☐	☐	☐	☐	☐

Please provide here any comments on answers to the previous questions you wish to clarify or expand:

....

....

....

....

....

....

Opinions

The following questions ask about your opinions regarding <u>palliative sedation **in paediatrics** as a means to alleviate severe suffering in the last hours to days of life</u>. “Continuous use" is either continuous subcutaneous/intravenous/transdermal administration or scheduled repeated administration with the intention of producing a continuous effect.

Please indicate to what extent you agree or disagree with the following statements.

	Strongly disagree	Disagree	Neutral / not sure	Agree	Strongly agree
19. I consider the continuous use of sedatives as a means to alleviate severe **<u>physical</u>** suffering **<u>in the last hours to days of life</u>** an acceptable medical practice.	☐	☐	☐	☐	☐
20. I consider the continuous use of sedatives as a means to alleviate severe **<u>psycho-existential</u>** suffering (in the absence of physical symptoms) **<u>in the last hours to days of life</u>** an acceptable medical practice.	☐	☐	☐	☐	☐
21. I consider the continuous use of sedatives as a means to alleviate severe **<u>physical</u>** suffering for patients who are expected to live for **<u>at least several weeks</u>** an acceptable medical practice.	☐	☐	☐	☐	☐
22. I consider the continuous use of sedatives as a means to alleviate severe **<u>psycho-existential</u>** suffering (in the absence of physical symptoms) for patients who are expected to live for **<u>at least several weeks</u>** an acceptable medical practice.	☐	☐	☐	☐	☐
23. I consider that a competent child with severe suffering after being fully informed has the right to demand the continuous use of sedatives in the last hours to days of life.	☐	☐	☐	☐	☐
24. I consider that parents of a child with severe suffering after being fully informed have the right to demand the continuous use of sedatives in the last hours to days of life	☐	☐	☐	☐	☐
25. I consider the continuous use of sedatives as a means to alleviate severe suffering in the last hours to days of life unnecessary, as suffering can always be relieved with other measures.	☐	☐	☐	☐	☐
26. I consider that the continuous use of sedatives in the last hours to days of life shortens the duration of the dying process.	☐	☐	☐	☐	☐
27. I feel that in clinical practice the continuous use of sedatives in the last hours to days of life can be difficult to distinguish from euthanasia.	☐	☐	☐	☐	☐
28. I consider routine withdrawal of artificial hydration/nutrition while providing the continuous use of sedatives as a means to alleviate severe suffering **<u>in the last hours to days of life</u>** acceptable.	☐	☐	☐	☐	☐
29. I consider routine withdrawal of artificial hydration/nutrition while providing the continuous use of sedatives as a means to alleviate severe suffering in patients who are expected to live for **<u>at least several weeks</u>** acceptable.	☐	☐	☐	☐	☐
30. I think that the continuous use of sedatives in the last hours to days of life cannot sufficiently alleviate suffering in all patients, even when patients become unresponsive.	☐	☐	☐	☐	☐
31. I think that dying in a sleep through the continuous use of sedatives can be a good death.	☐	☐	☐	☐	☐

Please provide here any comments on answers to the previous questions you wish to clarify or expand:

....
....
....
....
....
....

Your background

1. **What is your age group?**

   ☐ < 30 years

   ☐ 30 – 39 years

   ☐ 40 – 49 years

   ☐ ≥ 50 years

2. **What is your gender?**

   ☐ Female ☐ Male

   ☐ X ☐ Prefer not to say

3. **What is the clinical specialty you are working in <u>at this moment</u>?**
   (If more than one applies, please choose your main specialty.)

   ☐ Pediatric Palliative Medicine / Care
   ☐ Pediatrics (general)
   ☐ Pediatric Intensive Care
   ☐ Pediatric Oncology
   ☐ Pediatric Neurology
   ☐ Pediatric Cardiology
   ☐ Pediatric metabolic diseases
   ☐ Pediatric Anesthesiology
   ☐ Pediatric Surgery
   ☐ Neonatal Intensive Care
   ☐ Adult Palliative Care
   ☐ General / Family practice
   ☐ Other, namely: ________________

4. If general/family practice was indicated: does <u>your most recent experience</u> with palliative sedation cases relate to any of the following clinical specialties?

☐ Pediatric oncology
☐ Neonatology
☐ Pediatric metabolic diseases
☐ Pediatric neurology
☐ Pediatric cardiology
☐ Other, namely: ____________________________

5. **For how many years have you been working as a doctor?** _______ (years)

6. Are you a physician in training?

   ☐ Yes
   ☐ No

7. **Where are you working <u>at the moment</u>?**
   (Please choose <u>all answers</u> that apply.)

   ☐ University hospital
   ☐ Other hospital (non-university)
   ☐ Community palliative care services
   ☐ Home practice/family practice
   ☐ Inpatient hospice/palliative care unit
   ☐ Other, namely: ____________

8. **Did you receive a specialist palliative care training?**

   ☐ Yes, a training of < 1 day
   ☐ Yes, a training of 1-3 days
   ☐ Yes, a training of > 3 days
   ☐ No

9. **What do you consider to be your religion?**

☐ Christianity ☐ Islam

☐ Hinduism ☐ Buddhism

☐ Judaism ☐ No religion

☐ Other, namely: ________________ ☐ Prefer not to say

9b. **Do you believe that your religion or lack of religion has an impact on your medical practice?**

☐ Yes ☐ No

☐ I don't know ☐ Prefer not to say

10. **In the past 12 months for how many paediatric patients have you cared for in the last hours to days of life, either as primary physician or as consultant?**

(please estimate if you don't know the exact number)

☐ None
☐ 1-3 patients
☐ 4-6 patients
☐ >6 patients

END OF THE QUESTIONNAIRE
