# Supplementary material for: Physicians’ opinions on and practical experiences with palliative sedation therapy in children: an international survey in five European countries
Source: BMC Palliat Care. 2025 Oct 16;24:260. doi: 10.1186/s12904-025-01863-7 (PMC12532919; doi:10.1186/s12904-025-01863-7)
Supplement: Supplementary file 3 — Supplementary Material 3. [file 12904_2025_1863_MOESM3_ESM.pdf]

CASUS 1: PIETER – OSTEOSARCOMA

Pieter is a 10-year-old boy who complained of neck pain after a trauma; in the following month the pain increased and he lost his appetite, losing weight as a result. He has the feeling that he cannot swallow the food properly. Imaging shows a large tumour with almost complete destruction of vertebrae C1 and C2 with invasion of the base of the skull causing instability of the head and neck, stenosis of the spinal canal with limited pressure on the spinal cord and narrowing of the naso- and oropharynx. He is diagnosed with a localized osteosarcoma without distant metastases.

Therapy with cervical collar, analgesia and tube feeding is started as well as treatment of the osteosarcoma with chemotherapy. After an initial beneficial effect, a few months later there is pain again, with marked tumour progression on magnetic resonance imaging (MRI) scan. The tumour is inoperable and local radiotherapy is not curative with uncertainty whether this will improve his quality of life. Since there is no curative option the decision is made to focus on palliative care. Life expectancy is limited, but concerns months rather than weeks.

Tumour progression may lead to quadriplegia, severe arterial bleeding or endangered airway. Despite treatment with NSAID, opioids, Gabapentine and Amitriptyline, pain persists resulting in poor quality of life. Pieter says that ‘he wants to sleep and no longer wants to be in pain’.

CASUS 2: NORA – DEGENERATIVE METABOLIC DISEASE

Nora is a 6-year-old girl with leukodystrophy due to an inherited metabolic degenerative disorder. Prognosis in the late infantile form of the disease is usually death between the ages of 3 and 8 years, often due to severe pneumonia.

Because of increased swallowing problems and respiratory tract infections during the last few months with need for tube feeding and intermittent oxygen therapy at home, a do not resuscitate order was agreed with her parents six months ago, aiming to no longer starting intensive care (namely no non-invasive nor invasive ventilation, no resuscitation). Nora is now totally care-dependent, bedridden, and meanwhile blind. When Nora is not acutely ill, she is comfortable and enjoys her parents’ presence.

Nora arrives at the emergency department with her parents with fever, lethargy and severe shortness of breath. Her parents report that she is suffering continuous discomfort since three weeks due to stuck mucus that is difficult to cough up despite aspiration and physiotherapy at home. She is diagnosed with severe pneumonia with a sputum culture positive for Klebsiella aeruginosa, resistant to common antibiotics. Despite maximal non-invasive oxygen supplementation via a nasal canula, she still has severe dyspnoea. Nora is severely weakened and her chances to survive the current infection without intensive care are limited.

CASUS 3: LOUIS – PERIPARTAL ASPHYXIA

Louis was born by an emergency caesarean-section at 40 weeks 5 days, due to acute foetal distress with a tight umbilical cord. He suffered from severe peripartal asphyxia, with need for cardio-respiratory resuscitation, intubation and total body cooling. He started abnormal neurological behaviour and seizures, and received antiepileptic drugs several times during the following days. Electroencephalography and biochemical tests showed clear signs of severe asphyxia; Louis remained unresponsive and hypotonic.

A brain magnetic resonance imaging (MRI) on day 4 showed generalized, extensive hypoxic-ischemic lesions as well as involvement of the deep nuclei. A severe psychomotor disability, with inability to live independently, can be predicted with certainty. After team consultation and discussion with his parents, the decision is made to stop intensive care, and Louis is expected to die soon.

Louis is still receiving supportive ventilation, but he clearly has his own, albeit slow, breathing rhythm of about 20 breaths per minute. At this point, he is already receiving low-maintenance dose of morphine intravenously as pain relief during invasive ventilation. He shows few spontaneous movements but responds to painful stimuli. Louis is extubated in the presence of his parents.

Questions - all cases
1. Would you decide to initiate palliative sedation in this case at this moment?<br>☐ Yes<br>☐ No<br>☐ I don't know<br>☐ I do not wish to answer
2. Would you consult the parents regarding the decision to initiate palliative sedation?<br>☐ Yes, both parents must agree (incl. only parent and all other legal representatives)<br>☐ Yes, one parent must agree<br>☐ No, parental agreement is not necessary<br>☐ I don't know<br>☐ I do not wish to answer
3. Would you consult other healthcare providers regarding the decision to initiate palliative sedation? Multiple answers possible.<br>☐ Yes, during a multidisciplinary meeting<br>☐ Yes, consult with a physician from the team<br>☐ Yes, consult with a physician from the same hospital<br>☐ Yes, consult with a physician from another hospital<br>☐ Yes, consult with an expert in pediatric palliative sedation<br>☐ Yes, consult with a general practitioner<br>☐ Yes, consult with a paramedic (e.g. psychologist)<br>☐ No, consult is not necessary<br>☐ I don't know<br>☐ I do not wish to answer<br>☐ Yes, I would consult another healthcare provider, namely …
4. In case you would start palliative sedation, which medication(s) would you prefer to initiate palliative sedation in this case? Multiple answers possible.<br>☐ Midazolam (fast-acting benzodiazepine)<br>☐ Thiopental (fast-acting short-working benzodiazepine)<br>☐ Levomepromazine/Phenothiazine (dopamine-antagonist)<br>☐ Morphine (opioid)<br>☐ Fentanyl (opioid)<br>☐ Dexmedetomidine/Dexdor (intravenous anaesthetic)<br>☐ Propofol (intravenous anaesthetic)<br>☐ Clonidine/Catapres (central antihypertensive/sedative medication)<br>☐ Phenobarbital (barbiturate anticonvulsant)<br>☐ Other(s), namely …<br>☐ I don't know

☐ I do not wish to answer

5. Which of the following medical procedures would you perform along with initiation of palliative sedation, assuming there is parental agreement? Multiple answers possible.
   - ☐ Start or continue intravenous fluids
   - ☐ Discontinue intravenous fluids
   - ☐ Discontinue all medications that are not contributing to comfort
   - ☐ Discontinue feeding by tube or total parenteral nutrition
   - ☐ Discontinue cardiorespiratory monitoring during the last hours of life
   - ☐ I don’t know
   - ☐ I do not wish to answer

6. In your opinion, is it possible to initiate palliative sedation at home in this case, assuming all necessary healthcare providers and facilities are available?
   - ☐ Yes
   - ☐ No
   - ☐ I don’t know
   - ☐ I do not wish to answer

7. If palliative sedation was initiated, which treatment decisions would you consider appropriate in the following clinical situations?

The child is awake and comfortable. Which actions would you take?

- ☐ I do not change anything
- ☐ I increase the dose of the medication that was already started
- ☐ I increase the dose of the medication that was already started only if parents request to speed up the dying process
- ☐ I start additional medication, namely…
- ☐ I start additional medication only if parents request to speed up the dying process, namely…
- ☐ I don’t know
- ☐ I do not wish to answer
- ☐ Other(s), namely …

The child is completely unconscious and seems comfortable. Which actions would you take?

- ☐ I do not change anything
- ☐ I increase the dose of the medication that was already started
- ☐ I increase the dose of the medication that was already started only if parents request to speed up the dying process
- ☐ I start additional medication, namely…
- ☐ I start additional medication only if parents request to speed up the dying process, namely…
- ☐ I don’t know
- ☐ I do not wish to answer
- ☐ Other(s), namely …

The child shows signs of discomfort. Which actions would you take?

- ☐ I do not change anything

☐ I increase the dose of the medication that was already started
☐ I increase the dose of the medication that was already started only if parents request to speed up the dying process
☐ I start additional medication, namely…
☐ I start additional medication only if parents request to speed up the dying process, namely …
☐ I don't know
☐ I do not wish to answer
☐ Other(s), namely …

The child starts to gasp. Which actions would you take?

☐ I do not change anything
☐ I increase the dose of the medication that was already started
☐ I increase the dose of the medication that was already started only if parents request to speed up the dying process
☐ I start additional medication, namely …
☐ I start additional medication only if parents request to speed up the dying process, namely …
☐ I don't know
☐ I do not wish to answer
☐ Other(s), namely …

**** Extra questions – neonatal case ****

8. Would you adjust the low-dose morphine that was already started before extubating the neonate?
☐ Yes, stopping the morphine
☐ Yes, increasing the dose of morphine
☐ Yes, starting additional medication, namely…
☐ No
☐ I don't know
☐ I do not wish to answer

9. What would you do if, contrary to expectations, the neonate is comfortable (for a longer amount of time) and breathing independently after stopping intensive care, taking into account the poor prognosis in generalized hypoxic-ischemic encephalopathy?
☐ I stop the morphine because there is a chance of survival
☐ I continue the same dose of morphine and await further evolution
☐ I increase the dose of morphine
☐ I increase the dose of morphine only if parents request to speed up the dying process
☐ I start additional medication, namely …
☐ I don't know
☐ I do not wish to answer
☐ Other(s), namely …
